# Glucocorticoid suppression of osteocyte perilacunar remodeling is associated with subchondral bone degeneration in osteonecrosis

**DOI:** 10.1038/srep44618

**Published:** 2017-03-22

**Authors:** Tristan W. Fowler, Claire Acevedo, Courtney M. Mazur, Faith Hall-Glenn, Aaron J. Fields, Hrishikesh A. Bale, Robert O. Ritchie, Jeffrey C. Lotz, Thomas P. Vail, Tamara Alliston

**Affiliations:** 1 Department of Orthopaedic Surgery, University of California San Francisco, San Francisco, CA, USA; 2 Materials Science Division, Lawrence Berkeley National Laboratory, Berkeley, CA, USA; 3 UC Berkeley-UCSF Graduate Program in Bioengineering, University of California Berkeley, Berkeley, CA, USA; 4 Department of Materials Science and Engineering, University of California Berkeley, Berkeley, CA, USA

## Abstract

Through a process called perilacunar remodeling, bone-embedded osteocytes dynamically resorb and replace the surrounding perilacunar bone matrix to maintain mineral homeostasis. The vital canalicular networks required for osteocyte nourishment and communication, as well as the exquisitely organized bone extracellular matrix, also depend upon perilacunar remodeling. Nonetheless, many questions remain about the regulation of perilacunar remodeling and its role in skeletal disease. Here, we find that suppression of osteocyte-driven perilacunar remodeling, a fundamental cellular mechanism, plays a critical role in the glucocorticoid-induced osteonecrosis. In glucocorticoid-treated mice, we find that glucocorticoids coordinately suppress expression of several proteases required for perilacunar remodeling while causing degeneration of the osteocyte lacunocanalicular network, collagen disorganization, and matrix hypermineralization; all of which are apparent in human osteonecrotic lesions. Thus, osteocyte-mediated perilacunar remodeling maintains bone homeostasis, is dysregulated in skeletal disease, and may represent an attractive therapeutic target for the treatment of osteonecrosis.

Bone remodeling allows the skeleton to functionally adapt to changing mechanical and metabolic demands. Conversely, dysregulation of bone remodeling underlies osteonecrosis and many other skeletal diseases. Following extensive study of remodeling by bone forming osteoblasts and bone resorbing osteoclasts, bone-embedded osteocytes (OCYs) have emerged as crucial regulators of osteoblast and osteoclast differentiation and function^1^,^2^,^3^,^4^,^5^,^6^. However, until recently, the direct role of OCYs in bone matrix resorption was left largely unexplored^2^,^7^.

As early as 1910, OCYs were shown to actively participate in maintaining mineral homeostasis by resorbing their surrounding perilacunar bone matrix to release calcium and phosphate during metabolic stress due to rickets, hibernation, or avian egg production^8^,^9^,^10^,^11^,^12^. This cell-intrinsic bone-resorbing ability of OCYs, known as perilacunar remodeling^7^,^13^, was long overlooked^14^. Recent studies shed new light on this cellular mechanism by showing the induction of OCY-mediated perilacunar remodeling in lactating mice^15^, and its essential role in the maintenance of bone quality^16^. Furthermore, these studies show that OCY-mediated perilacunar remodeling is not only invoked in metabolic stress, but is also a constitutive homeostatic process^16^. Therefore, elucidating the role of perilacunar remodeling is pivotal to understanding the healthy skeleton and skeletal disease.

OCYs project dendritic processes through the intricate lacunocanalicular network to interact with one another, with other bone and marrow cells, and with the vasculature in the bone microenvironment^7^,^17^,^18^. The canalicular network deteriorates in mice deficient in proteases required for perilacunar remodeling, including MMP-2, MMP-13, and MMP-14^16^,^19^,^20^, suggesting an essential role for the activity of these enzymes. In addition, deficiency in these enzymes results in a profound collagen disorganization and matrix hypermineralization^16^. Not surprisingly, disruption of the mineralized collagen bone matrix compromises the material quality of bone, leading to increased bone fragility^16^,^21^. Because remodeling of the perilacunar matrix by MMPs helps to maintain the canalicular network and bone quality, we investigated the extent to which perilacunar remodeling is disrupted by skeletal disease.

In particular, we studied the role of perilacunar remodeling in glucocorticoid-induced osteonecrosis, also known as avascular necrosis^22^. Glucocorticoids (GCs) are a well-established risk factor for this intractable degenerative disease, which affects up to 20,000 Americans per year, with a median age of 41^23^,^24^,^25^,^26^,^27^. In osteonecrosis, subchondral bone insufficiency leads to disabling pain and eventual articular collapse, particularly in the femoral head^24^,^25^. Despite diverse clinical efforts to stimulate subchondral bone healing, osteonecrotic bone does not remodel or heal normally. Consequently, osteonecrotic joints almost always degenerate to the point that total joint replacement is required. Moreover, osteonecrosis is commonly bilateral and often presents as multi-focal disease^22^,^26^,^28^,^29^. However, therapies to prevent the progression of osteonecrosis to other joints of at-risk patients are not available. Given the importance of GCs as a therapy for rheumatologic diseases, asthma, and immunosuppression, new strategies to understand, prevent, and treat osteonecrosis are urgently needed.

A large body of research has elucidated mechanisms of GC action on bone, including GC effects on osteoblasts, osteoclasts, osteocytes, vasculature, bone mass, and bone quality^26^,^30^,^31^,^32^,^33^,^34^,^35^. Nonetheless, these mechanisms are insufficient to explain the bony sclerosis and subchondral bone fragility in GC-induced osteonecrosis. These features of osteonecrotic bone reminded us of the bony sclerosis and poor bone quality in MMP-13-deficient mice, which possess specific perilacunar remodeling defects^16^,^32^. However, the extent to which OCY-mediated perilacunar remodeling is sensitive to GCs or disrupted in osteonecrosis is unknown.

Therefore, since GCs can regulate OCYs and bone quality and represent the best-described risk factor contributing to osteonecrosis, we hypothesize that GCs alter OCY-mediated perilacunar remodeling in osteonecrosis. In addition to evaluating hallmarks of perilacunar remodeling in primary femoral heads from patients with GC-induced osteonecrosis, we determined if GC treatment was sufficient to alter functional outcomes of OCY perilacunar remodeling in mice. The identification in this study of defects in perilacunar remodeling in human osteonecrosis significantly advances the idea that perilacunar remodeling plays an important role in the development or progression of skeletal disease.

## Results

### GCs rapidly repress expression of genes required for perilacunar remodeling

**Figure 1:**
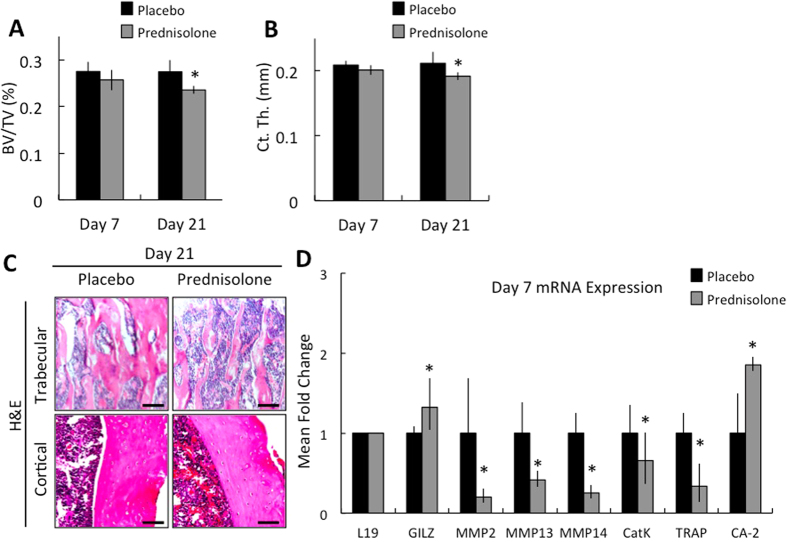
GCs specifically repress expression of enzymes required for OCY-mediated perilacunar remodeling. Bones from 2-month-old male FVB mice treated for 7 or 21 days with placebo or prednisolone were analyzed by μCT. (**A**) Bone volume over tissue volume (BV/TV) and (**B**) cortical thickness (Ct. Th.) were measured at the proximal femur and mid-shaft, respectively. (**C**) Histological sections of femurs stained with hematoxylin and eosin (scale bar = 50 μm) in proximal trabecular bone and mid-shaft cortical bone after 21 days treatment with placebo and prednisolone. Gene expression for GILZ, MMP2, MMP13, MMP14, CatK, TRAP, and CA-2 was evaluated in mRNA from marrow-free humeri extracted from mice after 7 days (**D**) of treatment with placebo or prednisolone, measured by RT-qPCR. All mRNA levels were normalized to ribosomal protein L19 mRNA levels and placebo treated animal expression. For all analyses, *p-value ≤ 0.05 vs. placebo-treated control, bars represent average ± SEM from n ≥ 6 animals for each group.

Using an established mouse model^36^,^37^,^38^, we evaluated the early cellular and molecular consequences of GCs on bone in mice treated with the GC prednisolone or vehicle for 7 or 21 days. Micro-computed tomography analysis confirms that prednisolone excess stimulated the anticipated reduction in trabecular bone volume and cortical thickness within 21, but not 7, days of treatment (Fig. 1A,B). Likewise, trabecular thinning was histologically apparent only after 21 days of prednisolone treatment (Fig. 1C, day 7 data not shown).

To examine the effect of GCs on OCY-mediated perilacunar remodeling, we evaluated in whole bone the dynamic effect of prednisolone on the expression of several genes that have been functionally implicated in perilacunar remodeling. Even before detectable differences in radiographic or histologic outcomes (Fig. 1A–C), 7 days of prednisolone rapidly and significantly repressed 5 of 6 genes required for perilacunar remodeling, including matrix metalloproteinases MMP-2, MMP-13, MMP-14, cathepsin K, and TRAP (Fig. 1D)^15^,^16^,^19^,^20^. The repression of MMP-2 and MMP-13 is sustained after 21 days of prednisolone treatment (data not shown). Therefore, significantly before the anticipated changes in trabecular bone volume or osteoclast function (Supplemental Fig. 1), the first change we detected is a strong, concerted repression of several genes required for OCY-mediated perilacunar remodeling.

### GCs alter OCY function independently of apoptosis

**Figure 2:**
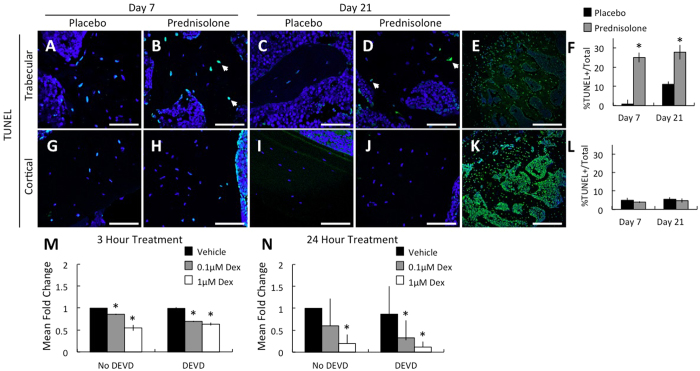
GCs induce a defect in osteocyte function independently of apoptosis in cortical bone. Histological sections of femurs stained for TUNEL activity (cells stained green, white arrows) and counterstained with Hoechst (scale bar = 50 um) in proximal trabecular bone (**A–D**) and mid-shaft cortical bone (**G–J**) after 7 and 21 days treatment with placebo and prednisolone. (**E**) Low magnification image of trabecular bone from Day 7 Prednisolone treated (scale bar = 200 um), and corresponds to section used for high magnification image shown in (**B**). (**F** and **L**), Average TUNEL-positive osteocytes over total osteocytes from 5 high powered fields per specimen, 4 specimens per group (Mean ± SEM, *p-value ≤ 0.05). (**K**) Positive control trabecular bone section treated with DNase (scale bar = 200 um). MLO-Y4 cells were treated with vehicle (Veh, PBS) or Dexamethasone (Dex) alone or after 30 minute pretreatment with Caspase-3 inhibitor, DEVD (1 μm). mRNA was isolated after 3 hours (**M**) and 24 hours (**N**) treatment and MMP13 expression measured by real-time quantitative PCR (n = 3 independent experiments, 3 biological replicates each). All mRNA levels were normalized to GAPDH mRNA levels and vehicle treated expression (*p-value ≤ 0.05).

GCs are well known to induce OCY apoptosis^33^,^34^,^35^,^39^,^40^,^41^,^42^,^43^,^44^. Accordingly, we observed more empty OCY lacunae in trabecular bone following 21 days of prednisolone treatment (Fig. 1C). However, the number of empty lacunae was unaffected by prednisolone in trabecular bone at 7 days, or at either time point in cortical bone (Fig. 1C, data not shown). Since the changes in perilacunar remodeling gene expression precede the appearance of empty lacunae, we evaluated the dynamic effect of prednisolone treatment on OCY apoptosis in each bone compartment. Using a TUNEL assay to detect DNA fragmentation, we observed a significant increase in OCY apoptosis in the prednisolone-treated trabecular compartment after both 7 and 21 days (Fig. 2A–F). The increased apoptosis in vehicle-treated mice at 21 days may reflect trabecular bone remodeling to generate marrow space following endochondral ossification in these skeletally immature mice^45^. However, OCYs in the cortical compartment showed no observable apoptosis at either time point (Fig. 2G–J,L), suggesting that trabecular bone OCYs are more susceptible to the apoptosis-inducing effects of GCs than those in cortical bone.

We next sought to determine if the GC-responsive changes in perilacunar remodeling gene expression were OCY-intrinsic and if they occurred independently of OCY apoptosis. To address this question, we examined the effect of glucocorticoids on MMP-13 expression in cultured MLO-Y4 OCY-like cells. Glucocorticoids rapidly and significantly repressed MMP-13 expression in a dose dependent (0.1 μM and 1 μM) manner within 3 hours (Fig. 2M); and that suppression became even more dramatic after 24 hours of treatment (Fig. 2N). Importantly, when we co-treated with DEVD, an inhibitor of caspase-3 activation, this rapid GC-dependent repression of MMP-13 remained. Trypan blue staining was used to confirm the ability of DEVD to prevent GC-induced apoptosis (Supplemental Fig. 2). Therefore, GCs repress MMP-13 in an OCY-intrinsic manner independently of OCY apoptosis. Since OCY apoptosis alone is insufficient to account for these GC-mediated effects on OCY, we hypothesize that GCs interfere with OCY perilacunar remodeling.

### Collagen disorganization shows perilacunar remodeling vulnerability to GCs

**Figure 3:**
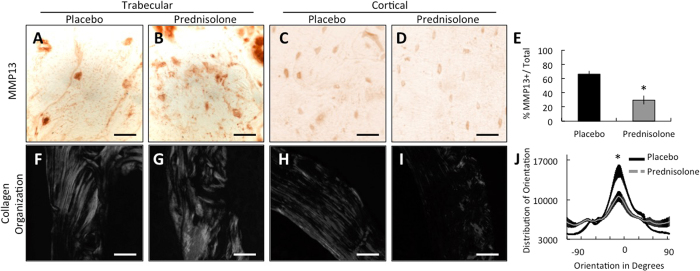
GCs repress hallmark outcomes of perilacunar remodeling in cortical bone. Immunohistochemistry for MMP-13 protein in placebo and prednisolone-treated trabecular (**A,B**) and cortical (**C,D**) compartments after 21 days treatment showing diminished staining in cortical bone due to GC treatment (scale bar = 20 *μ*m). (**E**) Quantification of percent MMP-13-postiive OCYs normalized to total OCYs in cortical bone. For all analyses, 5 high-powered fields per mouse, 4 mice per group (Mean ± SEM, *p-value ≤ 0.05 vs. placebo control). Representative picrosirius-red stained histological sections of the same bones viewed using polarized light microscopy show GC-dependent differences in collagen orientation in trabecular (**F,G**) and cortical (**H,I**) compartments (scale bar = 50 *μ*m), shown quantitatively for cortical bone in J. (**J**) Orientation J was used to determine the range of orientation degree for collagen and graphed as a composite average across multiple specimens per group. Each line represents the average ± SEM, *p-value ≤ 0.05 vs. placebo control peak orientation value.

We previously found that MMP-13-deficiency impairs the ability of OCYs to remodel the perilacunar bone matrix, resulting in collagen disorganization, hypermineralization, poor bone quality and degeneration of the lacunocanalicular network^16^. Evidence of defective perilacunar remodeling is also observed in mice deficient in MMP-2, MMP-14, or other enzymes^16^,^19^,^20^. Since GCs repress the expression of these factors in OCYs (Fig. 1D), we sought to determine if there is evidence of defective perilacunar remodeling in GC-treated mice. We evaluated bones from prednisolone-treated mice for the same hallmarks of defective perilacunar remodeling that we previously observed in MMP-13-deficient mouse bones. First, immunohistochemistry revealed lower MMP-13 protein expression levels and a significant reduction in the percentage of MMP-13-positive OCYs in trabecular and cortical bone following 21 days of prednisolone treatment (Fig. 3A–E). Furthermore, profound collagen disorganization was evident in both the trabecular and cortical bone following 21 days of prednisolone treatment (Fig. 3F–I). Quantitative analysis revealed a significant decrease in cortical bone collagen alignment, which was detectable after 21 days treatment of prednisolone (Fig. 3J). Unlike the localized collagen disorganization in mid-cortical bone of MMP-13-deficient mice^16^, the collagen disorganization in GC-treated mice is present throughout the cortical bone. This difference may result from the coordinated GC-mediated repression of multiple PLR enzymes, instead of only MMP-13, which is expressed at high levels in mid-cortical bone. Importantly, prednisolone-dependent repression of MMP-13 protein expression and collagen organization occurs even in cortical bone, where detectable OCY apoptosis was absent (Fig. 3D,I). Therefore, GC repression of perilacunar remodeling enzymes mimics MMP-13-deficiency, which causes collagen disorganization, hypermineralization, and bone fragility^16^,^46^.

### Hypermineralization develops rapidly following GC treatment

**Figure 4:**
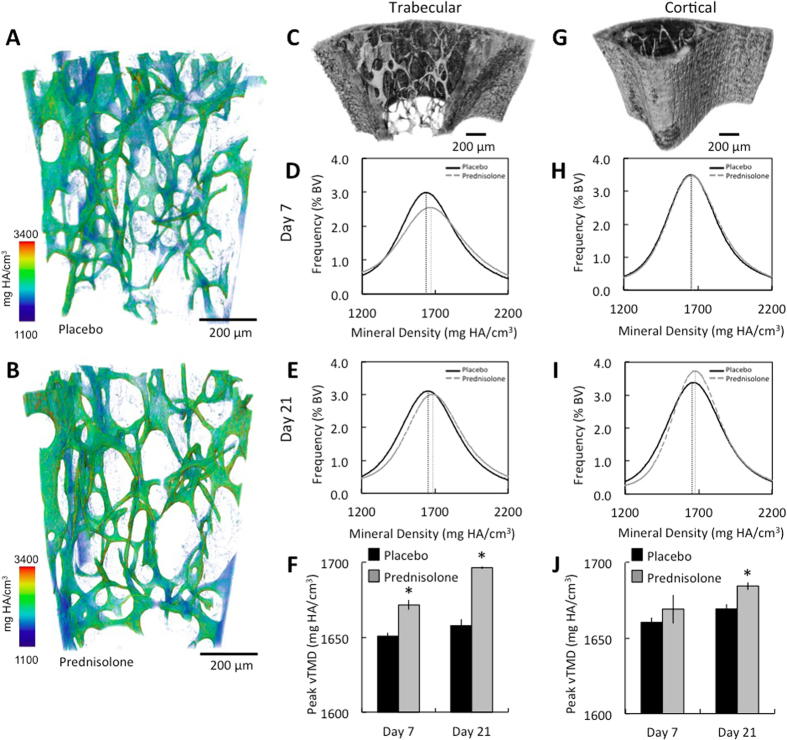
Hypermineralization develops rapidly and persists during GC treatment in cortical and trabecular bone. (**A,B**) Representative color scaled 3D rendered images of SRμT from trabecular bone of 7-day placebo (**A**) and prednisolone (**B**) -treated animals display a shift toward more highly mineralized bone with GC treatment. (**C,G**) Grey-scale 3D rendered images of SRμT highlight trabecular (**C**) and cortical (**G**) ROI that were analyzed quantitatively. Mineral density plotted against percent bone volume for day 7 (**D,H**) and day 21 (**E,I**) (dotted vertical lines correspond to peak mineral density) show significantly increased mineralization in prednisolone-treated bones, as quantified for trabecular (**F**) and cortical (**J**) bone. For all analyses, bars represent mean ± SEM *p-value ≤ 0.05, n ≥ 3.

To determine if bone matrix mineralization is also susceptible to the GC-dependent repression of MMP-13 and other perilacunar remodeling genes (Fig. 1D), we used synchrotron X-ray tomographic microscopy (SRμT). Key advantages of SRμT include its sensitivity and ability to quantitatively and qualitatively evaluate mineralization and structural features of bone ECM in 3D at the nanoscale (i.e., 1.3 μm resolution for this study). As shown colorimetrically (Fig. 4A,B), prednisolone treatment causes hypermineralization of mouse trabecular bone within 7 days. Quantitative analysis reveals a significant increase in trabecular bone matrix mineral density (mg HA/cm^3^) as early as 7 days after prednisolone treatment, an increase that persists throughout the 21-day period (Fig. 4C–F). Likewise, prednisolone significantly induced hypermineralization of cortical bone within 21 days (Fig. 4G–J), even in the absence of detectable OCY apoptosis (Fig. 2J). Therefore, as in MMP-13-deficient mice^16^, the GC-dependent repression of perilacunar remodeling enzyme expression rapidly disrupts both the mineral and organic phases of the bone matrix prior to OCY apoptosis.

### Rapid GC-dependent degeneration of the lacunocanalicular network

**Figure 5:**
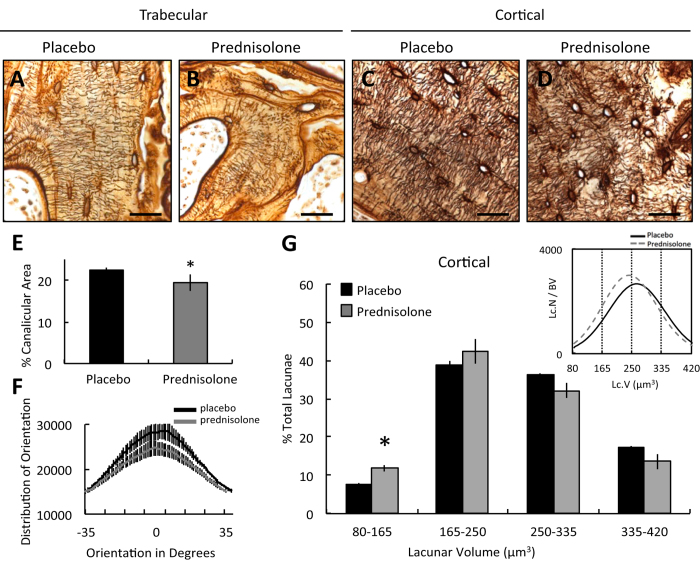
GCs repress hallmark outcomes of perilacunar remodeling in cortical bone. Representative silver nitrate-stained histological sections of femoral placebo (**A,C**) and prednisolone (**B,D**) treated bone after 21 days of treatment showing lacuno-canalicular network distribution and organization differences between trabecular (**A,B**) and cortical (**C,D**) compartments (scale bar = 20 *μ*m). (**E**) Quantification of average percent canalicular area (normalized to total area) and (**F**) distribution of canalicular orientation in cortical bone (**C,D**) after 21 days of treatment. XTM was used to determine osteocyte lacunar volume in cortical bone at day 7 (**G**). Inset graph represents mean lacunar volume plotted against lacunar number as a function of bone volume assessed. Vertical dotted lines signify the lacunar size ranges quantified in the bar graph (25% of the size range in each group). In addition to a shift of the overall lacunar size distribution in cortical bone at day 7, there is a significant increase in the overall percentage of smaller osteocytes due to prednisolone treatment. For all graphs, bars represent mean ± SEM from n ≥ 3, *p-value ≤ 0.05 compared to placebo control.

Another hallmark of defective perilacunar remodeling is degeneration of the lacunocanalicular network^15^,^16^,^19^,^20^,^46^. This network facilitates OCY cell-to-cell communication, enables transport of solutes, and connects the bone-embedded OCYs to bone’s nutrient-rich vascular supply^7^,^17^,^18^. Prednisolone caused a striking disorganization of canaliculi in both trabecular and cortical bone (Fig. 5A–D), along with a significant reduction in canalicular area (Fig. 5E) and orientation (Fig. 5F) in cortical bone. Although significant changes in the lacunocanalicular network were histologically apparent only after 21 days, synchrotron X-ray tomography (SRμT) offers the resolution required to quantify the volume of thousands of OCY lacunae throughout a 3D specimen. Using this approach, a shift toward smaller cortical bone lacunar size was detectable as early as 7 days after prednisolone treatment (Fig. 5G, inset). Prednisolone caused a significant increase in the number of small OCY lacunae (Fig. 5G). Importantly, the regulation of lacunar size appears to be dynamic, such that differences in lacunar size are not apparent in cortical bone after 21 days of GC treatment (Supplemental Fig. 3). This precedes the previously reported increase in lacunar size following 56 days of GC treatment^36^. Though many questions remain about the dynamics of perilacunar remodeling and the regulation of lacunar size, we find that GCs rapidly suppress resorption of the perilacunar bone matrix and ultimately impair OCY canalicular network connectivity, even prior to GC effects on OCY apoptosis.

### Dysregulation of bone architecture and mineralization in human osteonecrosis

**Figure 6:**
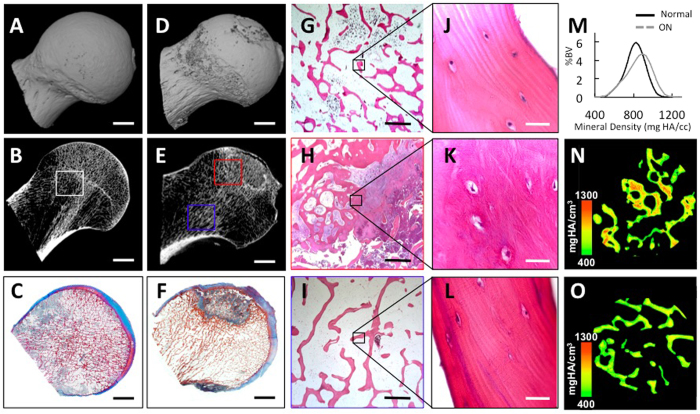
In human GC-induced osteonecrosis of the femoral head, trabecular bone matrix is hypermineralized and OCYs are viable. Three-dimensional reconstructions of a HR-pQCT scan of a cadaveric human femoral head from a donor without joint disease exhibits a smooth surface (**A**), organized trabecular microarchitecture and an intact cortical shell (**B**), white inset box signifies ROI in (**G** and **J**). Human osteonecrotic femoral head displaying fragmented surface (**D**), disorganized trabecular microarchitecture, and a thin cortical shell (**E**), red inset box signifies ROI in (**H**,**K** and **N**). Blue inset box signifies ROI in (**I**,**L** and **M**). Gross differences were apparent between Heidenhain’s trichrome-stained histologic sections of cadaveric (**C**) and osteonecrotic (**F**) femoral heads (Scale bar = 10 mm for (**A–F**). Hematoxylin and eosin-stained sections of cadaveric trabecular bone (**G,J**) and trabecular bone near (**H,K**) and far from (**I,L**) the osteonecrotic lesion shows lacunae occupied with OCYs in each condition (Scale bar = 200 *μ*m in (**G,H,I**) Scale bar = 20 *μ*m in (**J,K,L**). Quantitative (**M**) and qualitative (**N,O**) analysis of mineral density shows that trabeculae in the sclerotic zone of osteonecrotic lesions (**N**) are hypermineralized, relative to those distant from the lesion (**O**). In M, mineralization is normalized to percentage of bone volume within normal and osteonecrotic regions of interest (n = 3, see methods for details).

The ability of GCs to suppress PLR could play a casual role in osteonecrosis. To investigate this hypothesis, we collected human femoral heads following total hip arthroplasty surgery from patients with GC-induced osteonecrosis (Fig. 6D–F). These specimens were compared radiographically and histologically to age-matched cadaveric donor tissue (Fig. 6A–C) with no reported or apparent joint pathology. Three-dimensional radiographic reconstructions vividly reveal the failure of the subchondral bone plate in the osteonecrotic femoral head (Fig. 6D,E), which ultimately causes collapse of the overlying articular cartilage.

High-resolution peripheral computed tomography scans show the severe dysregulation of subchondral trabecular bone microarchitecture in osteonecrosis. Unlike the uniform trabeculae of the cadaveric femoral head (Fig. 6B,C), the osteonecrotic femoral head shows a range of trabecular phenotypes that represent the thin trabecular remnants within the necrotic lesion, surrounded by a ring of thick trabeculae in the sclerotic zone (Fig. 6E,F). Although empty OCY lacunae are prevalent within the necrotic lesion, the sclerotic zone contains no more empty lacunae than cadaveric trabeculae or trabeculae distant from the osteonecrotic lesion (Fig. 6J–L). Therefore, we focused on the bone in the sclerotic zone (Fig. 6E red box, H, K) and bone in the healthy regions (Fig. 6E blue box, I, L) where OCYs were still detectable.

Not only are the trabeculae of the sclerotic zone (Fig. 6E,F,H) thicker, but they are also hypermineralized relative to trabeculae from the equivalent region of a cadaveric femoral head (Fig. 6M–O). Comparison within the same specimen shows that the sclerotic bone around the osteonecrotic lesion is also significantly hypermineralized relative to apparently normal bone that is distant from the lesion (average of 2.4% increase in mineralization, p < 0.02). In contrast, micro-CT revealed no significant difference in bone matrix mineralization between the equivalent regions of cadaveric bone (See Supplemental Table 2). Thus, the mechanical failure of osteonecrotic subchondral bone is accompanied by profound changes in trabecular microarchitecture and bone matrix mineralization. The persistence of intact OCYs and the hypermineralization in sclerotic zone trabeculae are consistent with the apoptosis-independent effects of GCs on OCYs *in vitro* (Fig. 2M,N) and with the suppression of perilacunar remodeling and hypermineralization observed in GC-treated murine cortical bone where OCYs are still viable (Fig. 2G–J).

### Defective perilacunar remodeling in human GC-induced osteonecrosis

**Figure 7:**
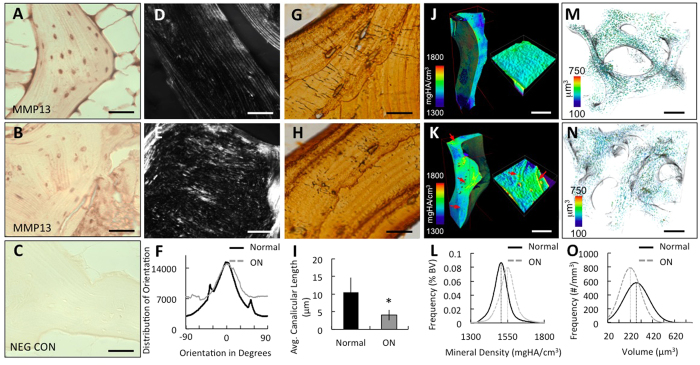
Organization and composition of human osteonecrosis subchondral bone has hallmarks of defective perilacunar remodeling. Trabeculae from the sclerotic regions of human osteonecrotic femoral heads (**B,E,H,K,N**) show histologic and radiographic hallmarks of defective perilacunar remodeling relative to trabeculae that are distant from the lesion (**A,D,G,J,M**). These include reduced MMP-13 expression as assessed by immunohistochemistry for MMP-13 or negative control (**A–C**) (scale bar = 20 *μ*m), defects in collagen organization as assessed using picrosirius red staining (**D–F**) (scale bar = 50 *μ*m), and reduced canalicular length as assessed by silver nitrate staining (**G–I**). For graph, bars represent mean ± SEM of n ≥ 3 regions from either normal human cadaveric or human osteonecrotic bone samples, *p-value ≤ 0.05 compared to control. As in GC-treated mouse bone, SRμT shows hypermineralization (**L**) (red arrows highlight hypermineralized, sclerotic, collagen layers) and reduced lacunar size (**O**) in the bone from human osteonecrosis lesions (**K,N**) relative to trabeculae that are more distant (**J,M**). Vertical dotted lines signify peak lacunar volume values.

Findings in GC-treated mice establish a causal relationship between GCs and perilacunar remodeling. As a next step in testing the hypothesis that GC-suppression of perilacunar remodeling plays a role in osteonecrosis, we evaluated perilacunar remodeling hallmarks in GC-induced human osteonecrosis. We compared trabeculae from sclerotic regions of osteonecrotic lesions to trabeculae that are distant from the lesions in the same femoral head of patients with GC-induced osteonecrosis. Remarkably, the sclerotic trabeculae exhibited the same characteristics of defective perilacunar remodeling that we observed in bone from mice treated with prednisolone for 21 days. Specifically, we observed a consistent qualitative repression of MMP-13 protein expression in the sclerotic trabeculae relative to normal trabeculae (Fig. 7A–C). As in GC-treated mice, GC-induced osteonecrosis in humans also disrupts both the organic and mineral phases of the bone matrix. Collagen in the sclerotic regions of osteonecrotic femoral heads is visibly disorganized with a shift in peak collagen fiber alignment relative to collagen fiber alignment in trabeculae distant from the lesions (Fig. 7D–F). The dramatic increase in bone matrix mineral density in the sclerotic trabeculae (Fig. 7L) results, in part, from streaks of highly mineralized bone matrix that are not detected in bone distant from the lesion (Fig. 7J,K). Finally, the lacunocanalicular network in sclerotic trabeculae is stunted and disorganized relative to trabeculae distant from the lesion or cadaveric bone (Fig. 7G,H). Histological analysis showed a significant 2-fold reduction in canalicular length in bone from these sclerotic regions (Fig. 7I). As in the GC-treated mouse bone (Fig. 4), SRμT analysis detected a dramatic shift toward smaller lacunar size in sclerotic bone from those regions near the osteonecrotic lesions (Fig. 7M–O). All of these perilacunar remodeling defects are observed in regions of bone that retained intact OCYs (Fig. 6J–L); consistent with evidence from mice that suppression of perilacunar remodeling occurs independently of OCY apoptosis. Collectively, these data identify a novel mechanism by which GCs compromise bone matrix integrity and OCY lacunocanalicular networks, as observed in human osteonecrosis, through repression of OCY perilacunar remodeling.

## Discussion

This study reveals that the homeostatic process of perilacunar remodeling, by which OCYs maintain bone extracellular matrix and lacunocanalicular networks, is vulnerable to GCs. Suppression of PLR is also evident in human GC-induced osteonecrosis. GCs drive a rapid and coordinated repression of a gene network required for osteocyte-mediated resorption of the perilacunar matrix, including MMP-13. This repression occurs in parallel with the known effects of GCs on osteoblasts, osteoclasts, and vasculature^23^,^25^,^26^,^47^, but prior to and independently of apoptosis. The exquisite sensitivity of SRμT quantitative analyses revealed that GCs rapidly decrease OCY lacunar size and induce matrix hypermineralization before the GC-dependent reduction in bone mass. Soon after, profound defects in the organization of collagen fibers and canalicular networks become apparent histologically. Human osteonecrotic bone phenocopied the characteristics of perilacunar remodeling suppression described in mice deficient in perilacunar remodeling enzymes such as MMP-2 or MMP-13^16^,^19^,^46^. Specifically, sclerotic bone around human osteonecrotic lesions displayed reduced MMP-13 expression, reduced lacunar size, degenerated canalicular networks, disorganized collagen fibers, and hypermineralization, which potentially underlie the mechanism for articular degeneration and collapse of osteonecrotic subchondral bone^48^. Not only do these findings provide insight into the cellular basis of GC-induced osteonecrosis, but they also extend the growing body of evidence that perilacunar remodeling is a rapid and dynamic process required for bone homeostasis.

GCs have previously been shown to induce and to repress MMP13 in bone and in other tissues^49^,^50^. The data presented here show that GCs coordinately and rapidly repress MMP2, MMP13, MMP14, and cathepsin K in OCYs, and that glucocorticoids repress perilacunar remodeling. Based on prior work by our group and others, which show the functional roles of MMP2, MMP13, MMP14, and cathepsin K in perilacunar remodeling, the observations herein support the idea that the coordinated repression of these enzymes by GCs is sufficient to repress perilacunar remodeling^16^,^19^,^20^. Nonetheless, additional studies will be needed to definitively establish this causal link.

Likewise, several lines of evidence indicate that GC suppression of perilacunar remodeling precedes apoptosis and is apoptosis-independent. These findings complement previous observations of increased osteocyte apoptosis and perilacunar hypomineralization in mouse bone after 56 days of GC treatment^18^,^32^,^34^. Within 21 days, we find that GCs repress MMP-13 protein expression in cortical bone in the absence of detectable osteocyte apoptosis (Fig. 2). *In vitro*, GCs induce OCY apoptosis or anoikis in a cell-attachment and Pyk2-dependent manner^34^. Using the same model system, we find that GCs robustly repress MMP-13 expression even when apoptosis is repressed with a Caspase-3 inhibitor. Several other features of suppressed perilacunar remodeling also appear prior to apoptosis. The most definitive data derive from murine cortical bone, which exhibits decreased lacunar size, lacunocanalicular and collagen disorganization, and hypermineralization; but no GC-induced apoptosis. Although osteocyte death in necrotic areas of human osteonecrotic lesions is well-defined^43^, the sclerotic trabeculae around these lesions have the same hallmarks of defective perilacunar remodeling with no apparent loss of osteocyte viability. We conclude that GCs can disrupt perilacunar remodeling independently of OCY apoptosis. Additional research is ongoing to determine the extent to which suppressed perilacunar remodeling, and the corresponding loss of lacunocanalicular circulation, plays a causal role in osteocyte apoptosis.

**Figure 8:**
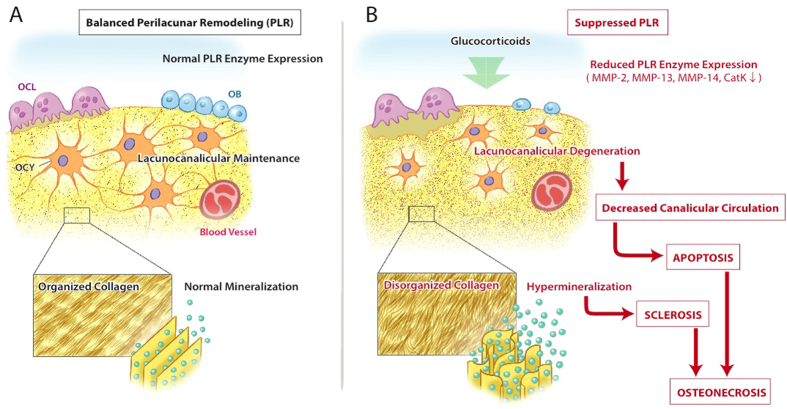
Schematic model comparing balanced perilacunar remodeling with glucocorticoid-mediated suppression. Within the bone matrix during normal perilacunar remodeling (**A**), there exists extensive lacunocanalicular connectivity, linearized collagen, and maintenance of mineral content around the osteocyte lacuna. Inset shows linearized collagen. In the presence of excess glucocorticoids (**B**), perilacunar remodeling is suppressed. Dysregulation of osteocyte enzyme expression likely precedes changes in bone matrix collagen alignment, lacunocanalicular circulation, and mineralization status; all of which contribute to the poor bone quality, sclerosis and increased fracture risk observed in osteonecrosis. Inset shows disorganized collagen matrix with accompanying hypermineralization. Illustration by M. Ouchida.

Nonetheless, we propose a hypothetical model (Fig. 8) by which GC repression of genes required for perilacunar remodeling first impairs the maintenance of lacunocanalicular networks and bone extracellular matrix integrity, followed by osteocyte apoptosis. This model adds insight to the cellular mechanisms contributing to GC-induced osteonecrosis — from a disease originating in the systemic vasculature — to one that begins with osteocytes and the canalicular circulation. In essence, the lacunocanalicular network forms the terminal end of the bone’s circulation that connects bone-embedded osteocytes to the systemic blood supply. Thus, dysregulation of osteocyte perilacunar remodeling by GCs disrupts OCY connectivity to the bone vascularity. This results in a phenotypically similar disease to that resulting from traumatic injuries to femoral arteries or blood clots in capillaries due to sickle cell anemia^26^,^51^, all of which have classically been called avascular necrosis^28^. These macroscopic similarities belie the need for distinct therapeutic approaches for each type of osteonecrosis and suggest that osteocytic perilacunar remodeling may be an attractive therapeutic target to prevent or delay the progressive development of bilateral disease. Further mechanistic studies are required to better understand, classify, and ultimately treat the osteonecrosis associated with these specific insults.

Discovery that perilacunar remodeling is disrupted in osteonecrosis sheds light not only on mechanisms by which glucocorticoid treatment could cause an ‘avascular’ phenotype and osteocyte apoptosis, but also on the signature subchondral bone fragility of osteonecrotic joints^22^. Perilacunar remodeling is essential for the maintenance of bone quality^15^,^16^. This was first shown in MMP-13-deficient mice, which had normal bone mass but significantly reduced work to fracture and fracture toughness^16^. As in MMP-13-deficient mice, bone from GC-treated mice and from sclerotic regions of the human femoral head is hypermineralized with disorganized collagen fibers. Indeed, others have observed hypermineralization due to defective OCY activity, such as in the model of ischemic osteonecrosis, where subchondral bone was described as ‘micropetrotic’^52^,^53^. Weinstein proposed that OCY apoptosis and mineralization are coupled, suggesting that inadequate canalicular circulation might also cause OCY apoptosis and subsequent hypermineralization^42^. Here we provide key evidence supporting this model to include the direct remodeling activity of OCYs. This study motivates additional investigation to directly test the causal role of osteocytic perilacunar remodeling suppression by GCs in osteonecrosis. Since OCY driven perilacunar remodeling is an essential homeostatic mechanism, its dysregulation may be implicated in other skeletal diseases. Indeed, perilacunar remodeling is regulated by PTH, sclerostin, and possibly other factors^15^,^54^. By showing the regulation of OCY-mediated perilacunar remodeling by GCs, and its disruption in osteonecrosis, this study supports the suggestion that osteocyte-mediated perilacunar remodeling may be an attractive therapeutic target for the treatment of skeletal disease^14^.

## Material and Methods

### Murine studies

All animal procedures described herein were performed according to national ethical guidelines and approved by the Institutional Animal Care and Use Committee (IACUC) at UCSF. In an established model, two-month-old male FVB mice were subcutaneously implanted with slow-release pellets containing placebo or prednisolone (2.8 mg/kg/d) (Innovative Research of America) and sacrificed at 7 and 21 days (n = 8/group)^32^.

### Murine bone micro-computed tomography

Micro-computed tomography of murine trabecular and cortical bone was performed using a Scanco μCT50 specimen scanner (Scanco) as previously described^16^,^46^,^55^. Briefly, bone specimens were harvested and fixed overnight in 10% neutral buffered formalin and transferred to 70% ethanol for scanning. A femoral region spanning the metaphysis and a femoral region spanning the mid-diaphysis were scanned. After scanning, scan projections were reconstructed to generate cross-sectional images using a cone-beam reconstruction algorithm. Density equivalent values are measured by calibration of the scanner to a hydroxyapatite phantom provided by the manufacturer.

### Human donor population and specimen preparation

Six subjects with a history of GC excess and with clinically diagnosed stage IV osteonecrosis of the femoral head, who were scheduled for total hip arthroplasty, were recruited for this study. All aspects of the work using tissues from human donors were performed in accordance with relevant guidelines and regulations. Specifically, recruitment of patients for donation of surgical wastes tissue occurred through referral from orthopedic surgeons at the University of California San Francisco (UCSF) Department of Orthopaedic Surgery. The UCSF Committee on Human Research approved the study protocol. Informed consent was obtained from each study participant prior to enrollment. Cadaveric tissue was obtained in accordance with the University of California – Policy Anatomical Donation/Materials Program through a protocol that was reviewed and approved by the UCSF Willed Body Program. Though most comparisons in human bone were made between the sclerotic zones of osteonecrotic lesions and bone distant from the lesion of the same femoral head, as an additional control, we also compared these findings to bone from femoral heads of a male cadaveric donor with no history of musculoskeletal or metabolic disease (Fig. 6A–C,G,J). This control specimen was obtained from the UCSF Willed Body Program. Each femoral head was removed as a single piece, HR-pQCT scanning performed and X-rays collected to document the severity of subchondral bone deterioration. Following imaging, femoral heads were cut into 7-mm-thick coronal slabs with a band saw for further analysis.

### High-resolution peripheral quantitative tomography of human femoral head

Osteonecrotic and cadaveric specimens were scanned with a high-resolution peripheral quantitative computed tomography system (HR-pQCT, XtremeCT, Scanco) to quantify mineral density. Orientations were adjusted so that the principal compressive trabeculae were oriented along the superior-inferior axis in coronal and sagittal views, and the fovea capitis faced medially in the axial view. Two parallel volumes of interest (VOI) were defined, each located at the mid-sagittal and mid-coronal positions of the femoral heads. The first VOI belonged to the sclerotic zone of the lesion, and encompassed the bone inferior to the fovea capitis. The second VOI appeared morphologically normal and was caudal to the first VOI. A visually determined fixed threshold was used to segment the hard tissue and marrow within the VOIs, and tissue mineral density was calculated by taking the mean hydroxyapatite density for all bone voxels within each VOI. For all analyses, regions of interest were selected from n = 6 femoral heads.

### Micro-computed tomography of human femoral head

To visualize the mineral heterogeneity, we evaluated bone cores using a benchtop micro-computed tomography (μCT) scanner, *μ*CT-40 (Scanco). For each of the 5 femoral heads examined, a mid-coronal slab was cored with a 6-mm diamond-tipped coring tool. Cores with locations approximating the VOIs defined using HR-pQCT were imaged. Attenuation values in the reconstructed images were converted to hydroxyapatite and mineral density normalized using a density calibration phantom. Tissue mineral density was calculated by taking the mean hydroxyapatite density for all bone voxels.

### Histology

#### Histology of murine specimens

For paraffin sectioning, dissected murine femurs were fixed in 10% neutral buffered formalin and incubated in 10% di- and tetra-sodium EDTA for 20–25 days until fully decalcified, followed by serial ethanol dehydration and paraffin embedding. Paraffin sections (7 *μ*m thick) were generated using a microtome (Leica) for polarized light microscopy, Ploton silver stain, tartrate-resistant acid phosphatase (TRAP) staining, TUNEL staining and immunohistochemistry, as described below^56^. For cortical bone, axial cuts were taken from the mid-diaphysis toward the distal condyles. For trabecular bone, sagittal sections were cut to include bone extending from the mid-diaphysis through the femoral head. Within these sections, analyses were performed in the cancellous compartment immediately distal to the lesser trochanter.

#### Histology of human specimens

Slabs were fixed in formalin, decalcified by ion-exchange (American Mastertech Scientific), dehydrated with ethanol, infiltrated with paraffin, sectioned, mounted on slides, and stained with a Heidenhain connective tissue stain containing aniline blue, orange G, and acid fuchsin. For all human histological analysis, data were collected from n = 3 specimens.

#### Histological analysis

For all histology, images were acquired using a Nikon Eclipse E800 bright-field microscope, unless otherwise noted. For all murine histological analysis, data were collected from n ≥ 4 femurs for each group. Each quantitative average represents 5 high-powered fields from each specimen.

Polarized light microscopy was performed on paraffin-embedded sections stained in a saturated aqueous solution of picric acid and 0.1% Direct Red-80 (aka: Picrosirius Red)(Sigma-Aldrich) as previously described^46^, then dehydrated, cleared, and mounted. Polarized filters were rotated to achieve the maximum birefringence. Birefringence was quantified using NIH ImageJ plug-in Orientation-J.

Ploton silver stain was used to visualize the lacunocanalicular network^56^. Paraffin-embedded sections were deparaffinized and rehydrated, then incubated in a solution of two-parts 50% silver nitrate and one-part 1% formic acid with 2% gelatin (Fisher Scientific) for 55 minutes. Stained slides were then washed in 5% sodium thiosulfate (Baker Chemicals) for 10 minutes and subsequently dehydrated, cleared, and mounted. Image J threshold application of gray-scale images resolved darker, silver stained lacunae and canaliculi. The resulting area and orientation was normalized to total bone area analyzed for each image captured and averaged. For human specimens, the total area of the lacunocanalicular network was negligible; therefore Image J was used to measure the average length of canalicular processes.

For TUNEL staining, sections were deparaffinized and treated with proteinase-K and 3% H_2_O_2_ for 5 minutes. Slides were incubated with *In Situ* Cell Death Detection kit (Roche) for 1 hour at 37 °C in the dark. Slides were washed and counterstained with Hoechst before being mounted with VectaMount (Vector). Apoptosis was visualized using a Leica DMi8 confocal fluorescent microscope and images collected using the Leica Application Suite X (LAS X). Quantification was performed using Image J cell counter to determine the average TUNEL-positive OCYs normalized to the total number of OCYs. For immunohistochemistry, slides were deparaffinized and hydrated prior to incubation in Innovex Unitrieve low temperature retrieval solution in a 60 °C water bath for 30 minutes. Endogenous peroxidase activity was quenched using 3% H_2_O_2_ for 10 minutes at room temperature. For all following steps, Innovex Universal Animal IHC kit was utilized. Background buster was applied for 30 minutes at room temperature. MMP-13 primary antibody was diluted 1:100 in PBS (Abcam #39012) and incubated in a humid chamber at 37 °C for 1 hour. Secondary linking antibody and horseradish peroxidase-enzyme were both used at room temperature for 10 minutes each. Fresh DAB solution was applied and incubated at room temperature for 5 minutes prior to wash with tap water and mounting with Innovex Advantage Mounting medium. Negative controls were performed by substituting Innovex Rabbit negative control sera in place of primary antibody. Quantification was performed using Image J cell counter to determine the average MMP-13-positive OCYs normalized to the total.

### RNA extraction and quantitative PCR

Humeri from placebo and GC-treated mice were dissected, the soft tissue, periosteum, and epiphyses removed prior to centrifugation to remove bone marrow. Bones were snap frozen and stored in liquid nitrogen until processing, as described^46^. Briefly, the frozen bones were homogenized using an Omni homogenizer in QIAzol Lysis Reagent to extract total RNA, and subsequently purified with RNeasy columns and on-column DNA digestion (Qiagen), according to manufacturer’s instructions. Quantity and 260/280 ratio of the extracted RNA were determined using a NanoDrop Spectrophotometer (Thermo Fisher Scientific). RNA was used to synthesize cDNA using the iScript cDNA Synthesis Kit (BioRad) according to manufacturer’s instructions. Gene expression was assessed using SYBR-based qRT-PCR with primers against *L19, Gilz, MMP-2, MMP-13, MMP-14, Ca-II, Ctsk, Trap, Opg, and Rankl* (See Supplemental Table 1 for primer sequences). Fold-induction was calculated using the delta-delta-CT method normalized to ribosomal protein L19^57^.

### Cell culture

The murine long bone–derived osteocytic cell line MLO-Y4 was generously provided by Dr. Lynda Bonewald. MLO-Y4 cells were cultured on collagen type I-coated plates (rat tail collagen type I, 0.15 mg/mL) in alpha minimum essential medium supplemented with 2.5% fetal bovine serum and 2.5% bovine calf serum in a 5% CO2 incubator at 37 °C as previously described^41^. DEVD was applied to respective wells to control caspase-3 cleavage for 30 minutes prior to addition of dexamethasone (Dex). After Dex treatment, parallel cultures were used to collect RNA after 3 and 24 hours. Analysis of RNA expression represents 3 biological replicates per treatment from 3 independent experiments as previously described^58^.

### Synchrotron X-ray computed micro-tomography

In order to visualize and quantify the 3-D lacunar volume and bone mineralization, bones were imaged by synchrotron x-ray computed micro-tomography (SRμT) at beamline 8.3.2 of the Advanced Light Source (ALS) (Lawrence Berkeley National Laboratory, Berkeley) as shown previously^59^. Scans were taken with a monochromatic x-ray energy of 20 keV with an exposure time of 800 ms, and 1025 projections were collected over a 180° rotation. A LuAG scintillator was used to convert x-rays in visible light that was imaged onto the camera with a 5X lens. The 5X images had a field of view of 3.3 mm horizontally and 2.8 mm vertically with a pixel size of 1.3 *μ*m. For each murine bone, one scan was taken in the trabecular region underneath the tibial metaphyseal growth plate and another one in the cortical region at the mid-diaphysis. Reconstruction of two-dimensional (2D) radiographs into three dimensions (3D) was performed using filtered back-projection with Octopus (Octopus v8). NIH ImageJ and Avizo (Visualization Sciences Group) were used to segment (binarization of the bone volume morphology), visualize and analyze the lacunar volume and the mineral density.

### Statistics

Data were analyzed by one-way ANOVA to determine significant differences between means of multiple groups. Tukey’s post-hoc analysis procedure was applied as indicated to determine which groups were significantly different from one another. For experiments involving the comparison of only two groups, Student’s t-test was used. P-values less than 0.05 were considered significant and are reported as such.

## Additional Information

**How to cite this article:** Fowler, T. W. *et al*. Glucocorticoid suppression of osteocyte perilacunar remodeling is associated with subchondral bone degeneration in osteonecrosis. *Sci. Rep.* **7**, 44618; doi: 10.1038/srep44618 (2017).

**Publisher's note:** Springer Nature remains neutral with regard to jurisdictional claims in published maps and institutional affiliations.

## Supplementary Material

Supplementary Information
