# Supplementary material for: Glucocorticoid suppression of osteocyte perilacunar remodeling is associated with subchondral bone degeneration in osteonecrosis
Source: Sci Rep. 2017 Mar 22;7:44618. doi: 10.1038/srep44618 (PMC5361115; doi:10.1038/srep44618)
Supplement: Supplementary Information [file srep44618-s1.pdf]

Supplementary Material

Glucocorticoid suppression of osteocyte perilacunar remodeling is associated with subchondral bone degeneration in osteonecrosis

Tristan W. Fowler[1], Claire Acevedo[1,2], Courtney M. Mazur[1,3], Faith Hall-Glenn[1], Aaron J. Fields[1], Hrishikesh A. Bale[2], Robert O. Ritchie[2,4,], Jeffrey C. Lotz[1,3], Thomas P. Vail[1], Tamara Alliston[1,3]

[1]Department of Orthopaedic Surgery, University of California San Francisco; [2]Materials Science Division, Lawrence Berkeley National Laboratory; [3]UC Berkeley/UCSF Graduate Program in Bioengineering, [4]Department of Materials Science and Engineering, University of California Berkeley

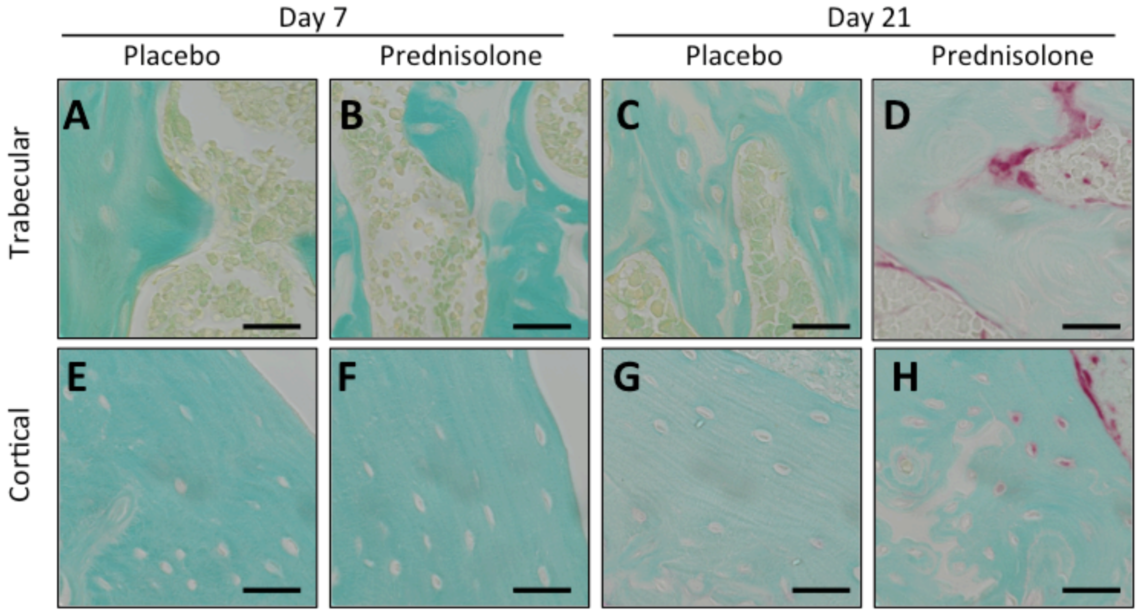

Supplemental Figure 1: GC treatment causes an increase in TRAP activity in osteoclasts and osteocytes after 21 days

Histological sections of femurs stained for TRAP expression (cells stained red) and counterstained with methyl green (scale bar = 50 um) in proximal trabecular bone (A-D) and mid-shaft cortical bone (E-H) after 7 and 21 days treatment with placebo and prednisolone. TRAP activity was absent in all samples except bone treated with prednisolone for 21 days. Interestingly, we observed large TRAP+ osteoclasts in the trabecular region (D) while the TRAP+ cells present in cortical bone appeared to be both osteoclasts and osteocytes.

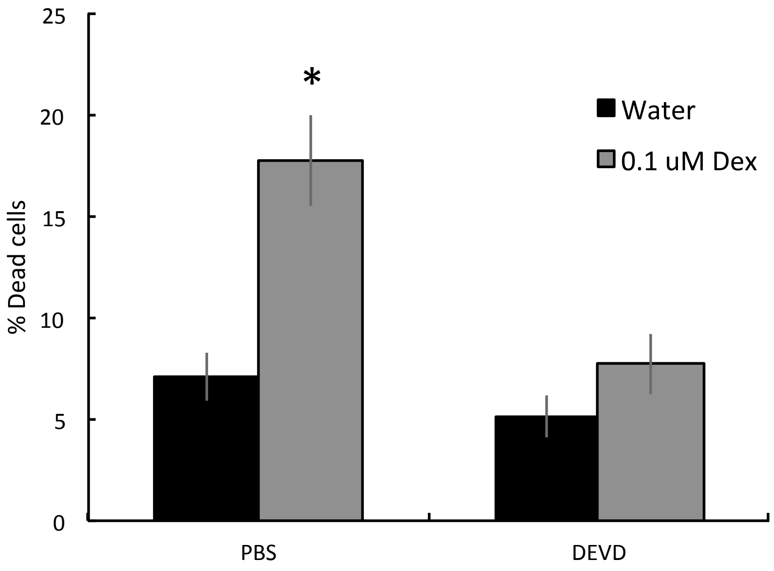

Supplemental Figure 2: DEVD blocks Dex-induced cell death

The ability of DEVD to block dexamethasone-induced apoptosis in osteocytes was determined as in Plotkin et al. MLO-Y4 osteocytic cells were cultured on collagen type I-coated dishes at 37 °C and 5% CO_2. To set up each experiment, cells were trypsinized, counted, and replated into collagen type I-coated 6-well plates at 50,000 cells/well. The next day, media was replaced and cells were pre-treated with 1 μM DEVD or vehicle (PBS) for 30 minutes. Dexamethasone (Dex) was dissolved in sterile water and applied to wells at a final concentration of 0.1 μM. An equal volume of vehicle (water) was used in control wells. All wells were harvested after 24 hours of Dex treatment.

To quantify cell death, nonadherent cells were collected from each well prior to trypsinization of cells adhered to the culture dish. The trypsinized cells were collected with media containing serum and combined with the nonadherent cells before centrifugation. Each cell pellet was resuspended in an equal volume of media and diluted in 0.4% trypan blue stain. Cells containing and excluding trypan blue staining were counted using a hemacytometer, with eight measurements made for each replicate of each condition.

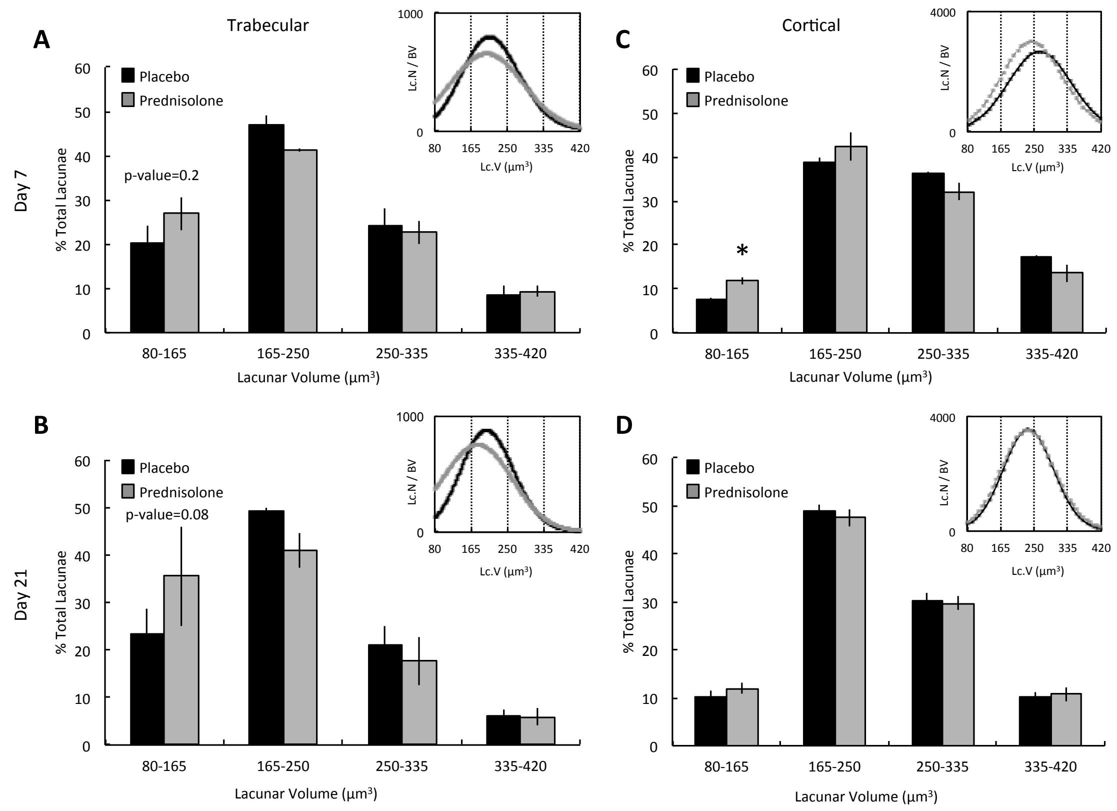

Supplemental Figure 3: GC treatment effects on lacunar volume

Graphs show the distribution of canalicular orientation in trabecular (A, B) and cortical bone (C, D) after 7 (A, C) and 21 (B, D) days of treatment. XTM was used to determine osteocyte lacunar volume. Inset graphs represent mean lacunar volume plotted against lacunar number as a function of bone volume assessed. Vertical dotted lines signify the lacunar size ranges quantified in the bar graph (25% of the size range in each group). In addition to a shift of the overall lacunar size distribution in cortical bone at day 7, there is a shift in the overall percentage of smaller osteocytes due to prednisolone treatment except in cortical bone after 21 days of treatment. For all graphs, bars represent mean ± SEM from $n \geq 3$, *=p-value$\leq$0.05 compared to placebo control.

Gene Name	RefSeq (mRNA)	F-sequence	R-sequence	Product length
MMP-2	NM_008610.3	AACGGTCGGGAATACAGCAG	GTAAACAAGGCTTCATGGGG	125
MMP-13	NM_008607.2	CGGGAATCCTGAAGAAGTCTACA	CTAAGCCAAAGAAAGATTGCATTTC	75
MMP-14	NM_008608.4	AGGAGACGGAGGTGATCATCATTG	GTCCCATGGCGTCTGAAGA	142
Gilz	NM_010286.4	TGACTGCAACGCCAAAGC	CTGATACATTTCGGTGTTCATGGTT	101
Rankl	NM_011613.3	CCAAGATCTCTAACATGACG	CACCATCAGCTGAAGATAGT	140
Car2	NM_009801.4	GAGCTTCACTTGGTTCACTGG	TGTGAGGCAGGTCCAATCTTC	113
Ctsk	NM_007802.4	GAGGGCCAACTCAAGAAGAA	GCCGTGGCGTTATACATACA	203
L19	NM_026490.2	ACGGCTTGCTGCCTTCGCAT	AGGAACCTTCTCTCGTCTTCCGGG	156

Supplemental Table 1: Primer sequences used for gene expression analysis; including the gene name targeted, RefSeq accession number, forward and reverse oligonucleotides, and expected product lengths.

Specimen Type	Sex	Age (years)	Normal (mean +/- SD) (HA/cc)	Sclerotic (mean +/- SD) (HA/cc)
**OSTEONECROTIC**			**ROI-1**	**ROI-2**
Right FH-Sample 1	Male	59	812.7 +/- 130.9	856.2 +/- 104.3
Left FH-Sample 1	Male	38	946.5 +/- 141	962.2 +/- 143.7
Left FH- Sample 2			944.9 +/- 152.7	956.3 +/- 147.7
Right FH-Sample 1			928.7 +/- 139	974.9 +/- 140.9
Right FH-Sample 2			899.3 +/- 145	921.8 +/- 129.5
Right FH-Sample 1	Male	56	954.8 +/- 157.8	949.7 +/- 142.4
Right FH-Sample 2			954.1 +/- 151.3	970.6 +/- 156.3

Specimen Type	Sex	Age (years)	Normal (mean +/- SD) (HA/cc)	Normal (mean +/- SD) (HA/cc)
**NO OSTEONECROSIS**			**ROI-1**	**ROI-2**
Left FH-Sample 1	Female	66	1015.8 +/- 156.5	997 +/- 157.2
Left FH-Sample 2			998.3 +/- 152.3	997.2 +/- 157
Left FH-Sample 1	Male	52	975.4 +/- 144.3	968.8 +/- 152.6
Left FH-Sample 2			994.5 +/- 150.5	994.5 +/- 153.5

Supplemental Table 2: Mineralization analysis from μCT scans of two separate regions of interest. For osteonecrotic samples, regions span from non-lesion to lesion. In the cadaveric controls, regions span comparable locations, with no pathological appearance of osteonecrosis. The side of isolation (left/right) is denoted for each patient. One patient, male 38, underwent bilateral arthroplasty.
